# Research on the influence of body image on college students’ weight-loss intention: chained intermediary analysis of self-efficacy and self-esteem

**DOI:** 10.3389/fpsyg.2024.1458259

**Published:** 2024-08-16

**Authors:** Ouyang Yiyi, Xiong Xue, Luo Jiong, Zhang Yanhui

**Affiliations:** ^1^School of Economics and Management, Chengdu Sport University, Chengdu, China; ^2^College of Physical Education, Chongqing University of Posts and Telecommunications, Chongqing, China; ^3^College of Physical Education, Southwest University, Chongqing, China

**Keywords:** body image, self-efficacy, self-esteem, weight-loss intention, intermediary effect

## Abstract

**Purpose:**

This paper aims to explore the relationship between body image, self-efficacy, self-esteem, and weight-loss intention among college students, offering insights to promote healthy and confident lifestyle habits.

**Methods:**

Undergraduate students from western China were selected using a stratified random sampling method. Data were analyzed using SPSS 19.0 and AMOS 21.0 statistical software.

**Results:**

(1) Body image showed a significantly positive correlation with self-efficacy and self-esteem but a negative correlation with weight-loss intention. Self-efficacy exhibited a significantly positive correlation with self-esteem and a negative correlation with weight-loss intention, while self-esteem was significantly negatively correlated with weight-loss intention. (2) Body image directly impacted weight-loss intention [Effect Size (ES) = −0.120]. Self-efficacy (ES = −0.069) and self-esteem (ES = −0.119) played significant mediating roles between body image and weight-loss intention, respectively. (3) The chained intermediary role of self-efficacy and self-esteem also reached significance (ES = −0.038).

**Conclusion:**

Body image conducts effect on the degree of weight-loss intention among college students through direct ways or indirect ways such as the intermediary role of self-efficacy and self-esteem, and also the chained intermediary role of self-efficacy and self-esteem. In addition, self-esteem is another key factors affecting college students’ weight-loss intention.

## Introduction

Body image refers to an individual’s cognitive picture of their physical appearance, comprising both objective perceptions and subjective evaluations of various bodily characteristics. This multifaceted cognitive picture encompasses dimensions such as appearance, body shape, physical abilities, and overall health condition. Importantly, the level of self-awareness regarding body image is known to significantly impact emotional states and health-related behaviors, such as weight control, social adaptation, stress management, personal development, and interpersonal interactions (Wang et al., 2017). In today’s world with highly developed online media, people often focus on their own body shape and appearance, while easily overlooking their health. Excessive exposure to styles like “slender figures” and “perfect bodies” publicized by media can lead to low body satisfaction. It is precisely because of this decline in body satisfaction that various weight-loss concepts emerge. Thus, some people may even resort to unhealthy weight-loss methods to improve negative body image. In this case, the risk of chronic diseases and psychological disorders is increased (Wei et al., 2012; Gillen, 2015; Andrew et al., 2016; Sun et al., 2017).

Weight-loss intention refers to various motivations that individuals derive when dissatisfied with their body shape such as the motivation to lose weight through exercise, dieting, or undergoing liposuction surgery at a cosmetic clinic (Huang et al., 2014; Lin and Lin, 2016). Relevant studies have found a strong correlation between individual body satisfaction and weight-loss intention. The more positive one’s body image, the lower their degree of weight-loss intention. Conversely, individuals dissatisfied with their body image or holding negative perceptions are more likely to initiate weight-loss intention and behaviors (Sun et al., 2017; Liu et al., 2019). Weight-loss behaviors come from weight-loss motivations and intention, both of which are then influenced by individuals’ cognitive processes, evaluations, and attitudes toward themselves. People with higher weight-loss motivation and intention are more likely to adopt extreme weight-loss methods and are at greater risk of exacerbating eating disorder symptoms (Ouyang et al., 2023). According to problem-behavior theory, an individual’s behavior should be studied from a developmental perspective and through the interaction of personality, environment, and behavior systems. An individual may exhibit different behaviors based on their environment, and similarly, the environment may have different effects on behavior depending on the individual (Jessor, 1987). Recent researches (Annesi, 2018; Hopkins and Bennett, 2018; Robertson et al., 2020) have pointed out that self-efficacy influences weight-loss intention, with higher self-efficacy associated with lower weight-loss intention, while lower self-efficacy associated with higher weight-loss intention. Self-efficacy refers to one’s subjective prediction of their abilities to accomplish a specific task. It also represents the tendency for individuals to judge and feel whether their actions can achieve a certain goal. It includes three meanings: firstly, it belongs to the category of ability perception but is not equivalent to actual abilities; secondly, it involves expectations about whether a particular goal can be achieved before engaging in an activity; thirdly, it means a subjective judgment of one’s ability to reach a goal (Ouyang et al., 2020). Meanwhile, some scholars have proposed that positive body image can effectively enhance self-efficacy, which in turn can mitigate and reduce the degree of weight-loss intention (Annesi and Mareno, 2015; Pan and Peña, 2017; Talleyrand et al., 2017; Soheila et al., 2018; Ouyang et al., 2021). This seems to have proved that body image may exert an influence on weight-loss intention through self-efficacy, potentially indicating an intermediary role of self-efficacy between body image and weight-loss intention.

Additionally, contemporary college students are particularly susceptible to internalizing the ideal “thin” body shape promoted by the media and the associated values. This makes them more likely to develop negative self-evaluations of their appearance, tending to view themselves as objects of others’ judgment, and neglecting their self-worth and internal feelings, which in turn leads to decreased self-esteem (Ouyang et al., 2021). Self-esteem refers to one’s positive or negative attitudes toward themselves, which can be both comprehensive and specific (Hülya et al., 2006). One may evaluate qualities of its own separately, or integrate these qualities to give an overall assessment. Therefore, self-esteem is an emotional assessment that individuals make about themselves, serving as a crucial indicator of whether health behaviors will be implemented. What’s more, it correlates well with the degree of weight-loss intention. Individuals with lower self-esteem often suffer from depression and anxiety, leading to an increase in weight-loss intention (Zhu et al., 2005; Margaret et al., 2017; Peng et al., 2017; Kamody et al., 2018). Based on relevant studies, body image is a significant factor influencing self-esteem among college students, with a positive predictive effect. Compared to college students with higher levels of body image, those with lower levels tend to spend more time on their bodies and comparing themselves with others. This behavior contributes to negative emotions and a decrease in self-esteem, thereby increasing weight-loss intentions among college students (Kamody et al., 2018; Ouyang et al., 2020). Furthermore, self-esteem is influenced by social values, the degree of importance attached to certain matters, and the evaluations individuals receive. Excessive social publicity of ideal body will strengthen college students’ focus on body shape, leading to an imbalance in their expectations of their bodies and then resulting in negative emotions and low self-esteem (Klomsten et al., 2004; Borges et al., 2010; Lian, 2012; Koronczai et al., 2013), driving an increase in weight-loss intention (Yigiter, 2014). Consequently, body image can influence weight-loss intention through self-esteem. To potentially reduce weight-loss intention among college students, it is important to both promote a healthy body image and address the improvement of self-esteem levels. This suggests that self-esteem may play an intermediary role between body image and weight-loss intention. At the same time, other studies have shown that self-efficacy has a positive influence on the self-esteem, which means individuals with higher self-efficacy tend to have stronger confidence (Cao and Zhang, 2018; Liao et al., 2019). Although self-efficacy primarily emphasizes an individual’s evaluation of their own abilities and self-esteem pertains to the evaluation of self-worth, enhancing self-efficacy can help increase a sense of control, thereby improving self-evaluation. Therefore, an increase in self-efficacy can enhance self-esteem. Further studies (Nock et al., 2016; Ouyang et al., 2023; Pyykkö et al., 2023) have demonstrated that as individuals’ self-efficacy improves, their self-esteem is likely to increase, which in turn reduces weight-loss intention.

In conclusion, body image, self-efficacy, and self-esteem are all significant factors influencing weight-loss intention, with each of these factors being important negative predictors of weight-loss intention. Body image can effectively enhance college students’ self-efficacy and self-esteem, thereby reducing their weight-loss intention. Moreover, there is a positive correlation between self-efficacy and self-esteem. This may indicate that positive body image of college students can enhance self-efficacy, leading to the development of stable self-esteem. Could this be a key factor in inhibiting weight-loss intention? However, previous research has rarely explored the relationship between “body image + self-efficacy + self-esteem + weight-loss intention,” particularly the lack of in-depth investigation into whether a chain mediating mechanism exists between “self-efficacy + self-esteem.” Based on this, our research constructs a chain mediation model of body image, self-efficacy, self-esteem, and weight-loss intention, and integrates body image, self-efficacy, and self-esteem as personality systems to examine their effects on weight-loss intention, to reveal the relevant mechanisms affecting weight-loss intention of college students and better promote the development of healthy and confident habits among college students, thus providing practical references for enhancing their physical and mental health. Therefore, the following hypotheses are proposed: (1) self-efficacy mediates body image and weight-loss intention; (2) self-esteem mediates body image and weight-loss intention; and (3) self-efficacy and self-esteem act as a chain mediator between body image and weight-loss intention.

## Subjects and methods

### Subjects

The survey was conducted with students enrolled in a comprehensive university in western China. First, the students were classified according to subject categories and grades, and then 1,000 students were randomly selected. With the help of physical education teachers and college counselors and the approval of the Ethics Committee, the selected subjects were investigated anonymously. A total of 1,000 questionnaires were distributed, and 887 valid questionnaires were recovered. The effective recovery rate of the questionnaire was 88.7%. The basic sample information is as follows (shown in Table 1): the participants include 472 male students and 415 female students with an average age of 20.91 ± 1.39 years, height of 172.19 ± 8.08 cm, weight of 65.88 ± 11.36 kg, and Body Mass Index (BMI) of 22.13 ± 2.92. This effective recovery rate was 82%. Among them, there are 234 freshmen, 322 sophomores, 245 juniors, and 197 seniors. The individual BMI values were converted according to BMI formula: BMI = weight (kilograms)/height (square meters). According to college students’ physical fitness BMI standard published by the Ministry of Education (Chen et al., 2019), the authors divided them into three groups by BMI: lightweight group (BMI < 20 kg/m^2^) with 217 samples, normal-weight group (BMI = 20–25 kg/m^2^) with 486 samples, and overweight group (BMI > 25 kg/m^2^) with 184 samples.

**Table 1:** Basic information of the sample (*N* = 887).

		*n*	%
Gender	Male	472	53.21
	Female	415	46.79
Grade	Freshmen	234	26.38
	Sophomore	211	23.79
	Junior	245	27.62
	Senior	197	20.74
BMI	Lightweight group	217	24.46
	Normal-weight group	486	54.79
	Overweight group	184	20.74

The average age is 20.91 ± 1.39 years, height of 172.19 ± 8.08 cm, weight of 65.88 ± 11.36 kg.

### Research methods

#### Questionnaire survey

This is a descriptive study with a structured questionnaire as the research tool, and all data are obtained from questionnaires. To ensure the preciseness and effectiveness of the research, a two-stage questionnaire survey approach was employed. Specifically, the procedure involved generating a preliminary draft based on the content of the research and the reference to a large number of research documents. Subsequently, a small-scale survey involving 200 participants was conducted, during which normality distribution, reliability analysis, and exploratory factor analysis were examined. Items with minimal contribution were eliminated, followed by structural model validation. Finally, the questionnaire is divided into five parts.

1. Personal background information: This component covers the basic background information on the subject’s gender, grade, height, weight, BMI, etc.
2. Body image scale: Mainly refers to the Multidimensional Body-Self Relations Questionnaire (MBSRQ) edited by Cash et al. with a total of 37 items (Cash et al., 1990). The scale is compiled by the Likert five-point scale. The options “Totally Agree,” “Very Agree,” “Not Sure,” “Very Disagree,” and “Totally Disagree” count as 5, 4, 3, 2, and 1 scores, respectively. The higher the total scores, the more positive is the perceived body image. The verification results of the measurement model of the scale are as follows: the parameter values of X^2^/DF = 1.539; Adjusted Goodness-of-Fit Index (AGFI), Comparative Fit Index (CFI), Tucker-Lewis Index (TLI), Incremental Fit Index (IFI), Goodness-of-Fit Index (GFI), and Root Mean Square Error of Approximation (RMSEA) are 0.945, 0.981, 0.987, 0.971, 0.976, and 0.049, respectively, which meets the acceptable standards and shows that the model fits well with the data obtained from the survey, and the scale has a good structure validity. The three dimensions are composed of physical fitness evaluation, weight concern, appearance adaptation, appearance evaluation, and health evaluation. Cronbach’s α values of the whole-body image scale and all its dimensions are greater than 0.70 (0.79–0.91), which fully confirms the good reliability of the scale. Therefore, the body image scale has good reliability and validity.
3. Self-efficacy scale: Mainly refers to the General Self Efficacy Scale (GSES) complied by Wang et al. (2001) with a total of 10 questions. The options “Totally Wrong,” “Basically Right,” “Almost Right,” and “Absolutely Right” count as 1, 2, 3, and 4 scores, respectively. The higher the total scores, the better is the general self-efficacy of an individual. The verification results of the measurement model of the scale are as follows: the parameter values of X^2^/DF = 1.609; AGFI, CFI, TLI, IFI, GFI, and RMSEA are 0.931, 0.928, 0.962, 0.954, 0.946, and 0.036, respectively, which meets the acceptable standards and shows that the scale has a good structure validity. Cronbach’s α values of the whole self-efficacy scale are equal to 0.82, which fully confirms the good reliability of the scale. Therefore, the self-efficacy scale has good reliability and validity.
4. Self-esteem scale: Mainly refers to the Self-esteem Scale complied by Rosenberg (Tian, 2006), and translated and revised to get 10 questions finally. The four options from “Totally Disagree,” to “Totally Agree” count as 1 to 4 scores, respectively, in which questions 2, 5, 6, 8, and 9 are reversed questions and will be scored in reverse. The verification results of the measurement model of the scale are as follows: the parameter values of X^2^/DF = 1.639; AGFI, CFI, TLI, IFI, GFI, and RMSEA are0.943, 0.991, 0.980, 0.991, 0.979, and 0.051, respectively, which meets the acceptable standards and shows that the scale has a good structure validity. The two dimensions are composed of self-acceptance and self-negation. Cronbach’s α values of the whole self-esteem scale and all its dimensions are greater than 0.70 (0.74–0.80), which fully confirms the good reliability of the scale. Therefore, the self-esteem scale has good reliability and validity.
5. Weight-loss intention scale: The weight-loss motivation subscale in the eating disorder inventory (EDI) adapted from Garner et al. (1983), with an additional statement from the author (“I am willing to lose weight”), totaling 6 questions. The subject expressed his/her weight-loss intention by scoring Likert five-level interval scale (5 = Strongly agree: 1 = Strongly disagree). The higher the score was, the stronger the weight-loss intention would be represented. The verification results of the measurement model of the scale are as follows: the parameter values of X^2^/DF = 1.561; AGFI, CFI, TLI, IFI, GFI, and RMSEA are 0.997, 0.994, 0.981, 0.992, 0.997, and 0.026, respectively, which meets the acceptable standards and shows that the scale has a good structure validity. Cronbach’s α values of the whole weight-loss intention scale are 0.73, which fully confirms the good reliability of the scale. Therefore, the weight-loss intention scale has good reliability and validity.

### Statistical analysis

Data obtained in this study was analyzed using SPSS19.0 and AMOS 21.0 software packages. The statistical methods included descriptive statistics, Kolmogorov–Smirnov test, reliability analysis, exploratory factor analysis, Harman single factor test, correlation analysis, structural equation model, Bootstrap analysis, etc. The significance level of all variables was set as α = 0.05.

To ensure the rigor of the research, before data analysis began, it was necessary to test the normal distribution of all variables in the pre-test and formal test. By using the Kolmogorov–Smirnov Test, it was determined that all the continuous variables in the pre-test and normal test conformed to a normal distribution (All the *p* values were significantly higher than 0.05).

This research uses Harman single-factor test to test the possible common method biases. The results show that the characteristic roots of a total of 14 factors are greater than 1, among which the largest factor explained variance is 21.11%, far from the critical standard of 40%. The research is less likely to be affected by common method biases, which is within the acceptable range.

## Results

### Related analysis on college students’ body image, self-efficacy, self-esteem, and weight-loss intention

Pearson correlation was used to analyze the correlation coefficients among body image, self-esteem, self-efficacy, and weight-loss intention (see Table 2). The results show that in the structure of physical fitness evaluation, appearance adaptation, appearance evaluation, weight concern, health evaluation, and self-esteem, self-acceptance and self-negation are positively correlated. Additionally, in this structure both self-acceptance and self-negation are all positively correlated with self-efficacy. However, self-acceptance and self-negation are all negatively correlated with weight-loss intention, while self-efficacy is inversely correlated with weight-loss intention. Furthermore, correlation analyses between demographic variables and research variables reveal that gender, grade, and BMI show no significant correlations with the variables of this research. Therefore, they are not controlled for in the hypothesis testing process. The results of the correlation analyses provide the foundation for testing subsequent hypotheses.

**Table 2:** Related analysis on college students’ body image, self-efficacy, self-esteem, and weight-loss intention (*N* = 887; *p < 0.05; **p < 0.01; ***p < 0.001).

	*M* ± SD	Gender	Grade	BMI	PFE	AD	AE	WC	HE	SA	SN	SE	WLI
Gender	–	1.00											
Grade	–	0.01	1.00										
BMI	22.13 ± 2.92	0.01	0.02	1.00									
PFE	29.20 ± 9.90	0.03	0.04	0.03	1.00								
AD	10.71 ± 4.08	0.04	0.01	0.01	0.57^***^	1.00							
AE	13.62 ± 5.27	−0.03	0.02	0.01	0.53^***^	0.48^***^	1.00						
WC	10.62 ± 3.95	−0.02	0.03	0.03	0.52^***^	0.47^***^	0.54^***^	1.00					
HE	13.31 ± 4.01	0.01	0.01	0.04	0.43^***^	0.49^***^	0.46^***^	0.54^***^	1.00				
SA	13.11 ± 2.89	0.01	0.03	0.03	0.23^***^	0.25^***^	0.33^***^	0.19^***^	0.25^***^	1.00			
SN	15.63 ± 2.84	0.01	0.02	0.01	0.22^***^	0.21^***^	0.24^***^	0.23^***^	0.17^***^	0.55^***^	1.00		
SE	24.91 ± 5.68	−0.02	0.03	0.01	0.27^***^	0.24^***^	0.22^***^	0.29^***^	0.28^***^	0.31^***^	0.30^***^	1.00	
WLI	13.90 ± 5.72	0.04	0.03	0.01	−0.28^***^	−0.20^***^	−0.35^***^	−0.24^***^	−0.26^***^	−0.33^***^	−0.39^***^	0.37^***^	1.00

PFE, physical fitness evaluation; AD, appearance adaptation; AE, appearance evaluation; WC, weight concern; HE, health evaluation; SA, self-acceptance; SN, self-negation; SE, self-efficacy; WLI, weight-loss intention.

### Model validation analysis of college students’ body image, self-efficacy, self-esteem, and weight-loss intention

**Figure 1:**
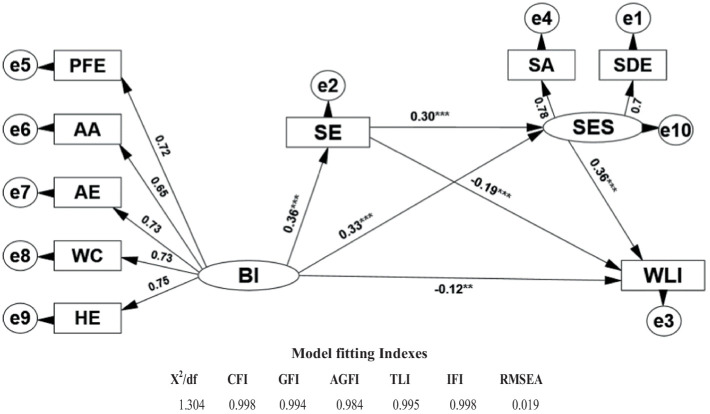
Path analysis diagram and model fit test of body image, self-efficacy, self-esteem, and weight-loss intention. PFE, physical fitness evaluation; AD, appearance adaptation; AE, appearance evaluation; WC, weight concern; HE, health evaluation; SA, self-acceptance; SN, self-negation; SE, self-efficacy; WLI, weight-loss intention; BI, Body image; SES, self-esteem; SE, self-efficacy.

To investigate the relationship between body image and self-esteem, self-efficacy, and weight-loss intention and examine the intermediary role of self-esteem and self-efficacy and according to the intermediary effect testing process proposed by Wen and Ye (2014), this research adopts AMOS to make a structural equation model analysis on the relationship among body image, self-efficacy, self-esteem, and weight-loss intention. Take for example Figure 1 after the model has been modified, and the model fit indexes are: X^2^/DF = 1.304 < 2.000, CFI = 0.998, GFI = 0.994, AGFI = 0.984, TLI = 0.995, and IFI = 0.998, all of which are >0.900 and RMSEA = 0.019 < 0.080, showing that the model can be built up.

From the findings, it is evident that from the standardized path coefficient β and significance level in the mixed model structure in Figure 1 that body image has a significantly positive predictive effect on self-efficacy and weight-loss intention (β = 0.36^***^, *p* < 0.001; β = 0.33^***^, *p* < 0.001), while body image has a significantly negative predictive effect on weight-loss intention (β = −0.12^**^, *p* < 0.01). Additionally, both self-efficacy and self-esteem demonstrate significant negative predictive effects on weight loss intentions (β = −0.19^***^, *p* < 0.001; β = −0.36^***^, *p* < 0.001, respectively). Moreover, self-efficacy positively predicts self-esteem significantly (β = −0.30^***^, *p* < 0.001). To verify the intermediary effect of self-efficacy and self-esteem on body image and weight-loss intention, the research adopts a non-parametric percentile Bootstrap process to make a significance test on intermediary effect. Five thousand samples were taken repeatedly from the original data to calculate a 95% Confidence Interval (CI). If the standardized path coefficient 95% CI does not include 0, that means the intermediary effect is significant. The 95% CI of the chained intermediary effect of body image from self-efficacy, self-esteem, to weight-loss intention is from −0.049 to −0.020; that of body image from self-efficacy to weight-loss intention is from −0.099 to −0.047; that of body image from self-esteem to weight-loss intention is from −0.152 to −0.076. The three intervals mentioned earlier do not include 0, which shows each intermediary effect is significant. Further decomposing the effects of each variable on weight-loss intention, as shown in Table 3, the direct effect of body image on weight-loss intention is −0.120. The total intermediary effect (−0.226) is the sum of the intermediary effects of the three paths, which represents the total indirect effect. The sum of the direct effect and the total intermediary effect is the total effect, which is −0.346. The effect sizes of the three intermediary paths in this research are calculated by dividing each intermediary effect value by the total effect. They are 10.98, 19.94, and 34.39%, respectively.

**Table 3:** Path and effect decomposition table of body image on weight-loss intention.

Effect	Path relationship	Effect size	Bootstrap SE	Bootstrap 95%CI	Relative intermediary effect (%)
Direct effect	Body image→weight-loss intention	−0.120	0.026	[−0.175, −0.071]	34.68
Indirect effect	Body image→self-efficacy→self-esteem→weight-loss intention	−0.038	0.007	[−0.049, −0.020]	10.98
	Body image→self-efficacy→weight-loss intention	−0.069	0.013	[−0.099, −0.047]	19.94
	Body image→self-esteem→weight-loss intention	−0.119	0.019	[−0.152, −0.076]	34.39
Total intermediary effect		−0.226	0.031	[−0.280, −0.156]	65.32

## Discussion

### Influence of body image on weight-loss intention

This research finds that body image has a significantly negative predictive effect on weight-loss intention. This result indicates that individuals with more positive body image tend to have lower weight-loss intention, which is consistent with the findings of scholars such as Sun et al. (2017) and Liu et al. (2019). Related research suggests that college students are easily exposed to social and cultural pressure and tend to internalize the ideal images promoted by the media, using these media standards as their own. Excessive exposure to ideal body image can lead to lower body image satisfaction. Those who do not meet these standards are likely to develop varying degrees of weight-loss intention and engage in weight-loss behaviors (e.g., dieting, taking weight-loss products, excessive exercise) to conform to these standards (Hou and Lu, 2008; Wei et al., 2012; Kvardova et al., 2020; Marks et al., 2020). In addition, influenced by factors such as family, peers, and media, weight-loss behaviors have become a trend and fashion. Slim and slender figures are highly favored and acknowledged by adolescents, leading them to adopt various weight-loss methods in pursuit of the ideal slim body shape. However, unreasonable weight-loss methods can cause both physical and psychological harm to adolescents. Furthermore, some scholars argue that adolescents tend to have a cognitive bias between actual body weight and ideal body weight. They often perceive their actual body weight to be heavier than their ideal body weight, leading to a heightened focus on appearance and the pursuit of a rational slim body shape and body image. This ultimately results in a decrease in adolescents’ body image and an increase in obesity anxiety (Sun et al., 2013; Cao et al., 2014; Huang and Liu, 2019). Liu et al. (2019) also believed that body image can negatively predict the degree of weight-loss intention among college students. The lower college students’ satisfaction with their body image, the higher their weight-loss intention. Therefore, body image is an important factor influencing the degree of weight-loss intention among college students, and there is a negative predictive effect between body image and weight-loss intention.

### Intermediary effect of self-efficacy

This research finds that body image has a significant positive effect on self-efficacy. This result indicates that people with more positive body image tend to have higher self-efficacy, whereas those with more negative body image tend to have lower self-efficacy. This result is basically consistent with the findings of the research of Stockton et al. (2009) and Ouyang et al. (2020). Meanwhile, studies have also confirmed that college students’ body image can positively predict the level of self-efficacy, and the more positive the body image of college students, the higher their self-efficacy (Wu et al., 2006; Annesi, 2019). This study also finds that self-efficacy has a significant negative effect on the weight-loss intention, which indicates that the higher the self-efficacy of college students, the lower the weight-loss intention. Indeed, it proves the research findings of Annesi, Hopkins and Robertson that there is a significant negative correlation between self-efficacy and weight-loss intention, with college students who have higher self-efficacy tending to have lower weight-loss intention, and those with lower self-efficacy tending to have higher weight-loss intention (Annesi, 2018; Hopkins and Bennett, 2018; Robertson et al., 2020). This study also finds that body image can negatively affect weight-loss intention both directly and indirectly through the intermediary effect of self-efficacy, indicating that a positive body image can enhance one’s cognitive evaluation of their body, thus promoting self-efficacy among college students (Zhang et al., 2015). As self-efficacy increases, individuals tend to have more positive self-evaluations, effectively improving college students’ perceptions and evaluations of their own body image, leading to increased self-acceptance and subsequently reducing weight-loss intention (Annesi and Mareno, 2015; Pan and Peña, 2017; Talleyrand et al., 2017; Soheila et al., 2018; Ouyang et al., 2021). Therefore, body image can influence weight-loss intention through self-efficacy.

In conclusion, combined with the Bootstrap test program for intermediary effect, the first hypothesis that self-efficacy plays an intermediary role between body image and weight-loss intention is true. Therefore, in order to promote the correct and positive body image, college students can not only directly improve their weight-loss intention, but also indirectly affect their weight-loss intention through increasing self-efficacy.

### Intermediary effect of self-esteem

This research finds that body image has a significant positive effect on self-esteem, indicating that the more positively individuals perceive their body image, the higher their self-esteem tends to be, and vice versa. This is consistent with the findings of Borges, Guo, Peng, and Zheng: body image has a significant positive predictive effect on self-esteem (Borges et al., 2010; Guo et al., 2017; Peng et al., 2017; Zheng et al., 2019). Scholars such as Kamody, Koronczai, Leng and Zhang argue that adolescent body image is a crucial factor influencing self-esteem (Koronczai et al., 2013; Zhang et al., 2015; Kamody et al., 2018; Leng et al., 2020). Moreover, body image serves as a reliable predictor of both physical self and self-esteem. Positive body image tends to be associated with a heightened sense of body value and positive emotions, which can enhance adolescents’ self-esteem, confidence, and self-acceptance. Conversely, negative body image is linked to a diminished sense of body value and extreme depressive emotions, leading to lower self-esteem, lack of confidence, and self-negation. Furthermore, college students are more likely to be influenced by societal values, the importance they place on certain issues, and evaluations. Excessive societal promotion of ideal body standards increases their focus on body shape, leading to an imbalance between their body expectations and reality, resulting in negative emotions and low self-esteem (Christine et al., 2020; Ouyang et al., 2023). This study also finds a significant negative impact of self-esteem on weight-loss intention. Individuals with low self-esteem tend to pay more attention to their body shape, leading to stronger weight-loss intentions. Conversely, those with high self-esteem feel more confident and acceptive of their body shape, resulting in reduced weight-loss intention. These research results have just proved those of Bruin, Lai, and Lei, who suggest that self-esteem is an emotional evaluation individuals have of themselves, serving as a crucial factor in predicting the implementation of health behaviors (Lei et al., 2005; Lai et al., 2006; Bruin et al., 2009). Research also suggests that self-esteem is a crucial factor in mitigating weight-loss intention among college students. Those with high self-esteem are more confident in their body shape, which reduces their weight-loss intention, whereas those with low self-esteem are less confident and thus have a stronger weight-loss intention. Therefore, the level of self-esteem in college students can effectively reflect the degree of their weight-loss intention (Margaret et al., 2017; Cao and Zhang, 2018; Kamody et al., 2018; Christine et al., 2020; Selensky and Carels, 2021). This study also finds that body image can indirectly influence weight-loss intention through the intermediary effect of self-esteem. Klomsten and Lian agree that body image is the most powerful predictor of overall self-esteem that in turn negatively impacts weight-loss intention (Lian, 2012; Koronczai et al., 2013). Meanwhile, research has also confirmed that an increasing number of college students, due to dissatisfaction with their body shape, often experience depression, anxiety, and negative body image, which leads to a decrease in self-esteem. This dissatisfaction drives them to alter their body image to align with current esthetic standards, thereby increasing their weight-loss intention (Yigiter, 2014). Conversely, college students with a more positive body image typically have higher self-esteem and lower weight-loss intention (Margaret et al., 2017; Peng et al., 2017; Christine et al., 2020; Selensky and Carels, 2021). Therefore, body image can influence weight-loss intention through self-esteem.

In conclusion, combined with the Bootstrap test program for intermediary effect, the second hypothesis that self-esteem plays an intermediary role between body image and weight-loss intention is true. Positive body image among college students can enhance self-esteem, and stable self-esteem plays a crucial role in inhibiting weight-loss intention. Therefore, in addition to directly improving weight-loss intention, body image can also indirectly influence them by increasing self-esteem.

### Chained intermediary effect of self-efficacy and self-esteem

This research shows through the results of the Bootstrap test program for the intermediary effect that body image can affect college students’ weight-loss intention through self-efficacy, which affects through part of the intermediary effect of self-esteem. This verifies Hypothesis 3 that self-efficacy and self-esteem play a chained intermediary role between body image and weight-loss intention, showing that individuals with lower body image are more likely to have lower self-efficacy, leading to lower self-esteem and even self-negation in promoting the college students’ weight-loss intention. On the contrary, individuals with higher body image tend to enhance self-efficacy among college students, thereby boosting self-esteem and fostering a stronger self-acceptance, which in turn can effectively suppress weight-loss intention among college students. As previous research has already confirmed, both self-efficacy and self-esteem serve as mediators between body image and weight-loss intention. Furthermore, previous studies have proved the correlation between self-efficacy and self-esteem, indicating that higher levels of self-efficacy positively influence an individual’s self-esteem, while lower levels of self-efficacy may hinder it (Wang et al., 2016; Ouyang et al., 2021). Additionally, scholars have found (Nock et al., 2016; Ouyang et al., 2023; Pyykkö et al., 2023) that as college students’ self-efficacy increases, it positively promotes their self-esteem, which in turn reduces weight-loss intention. This supports the fact that an improvement in body image among college students can enhance their self-efficacy, leading to stronger self-esteem and a subsequent reduction in weight-loss intention. Conversely, a decline in body image has the opposite effect. Therefore, college students’ body image negatively impacts their weight-loss intention through the chain mediation effect of self-efficacy and self-esteem.

In conclusion, body image can directly impact weight-loss intention, as well as indirectly influence them through the intermediary effects of self-efficacy, self-esteem, and the chained intermediary effect of self-efficacy and self-esteem. These findings provide theoretical foundations for establishing a positive body image among college students. Meanwhile, they offer important practical insights for promoting self-efficacy and self-esteem, and reducing unhealthy weight-loss behaviors among college students.

## Conclusion and limitation

### Conclusion

Body image has a significantly positive correlation on self-efficacy and self-esteem, and has a significantly negative correlation on weight-loss intention; self-efficacy has a significantly positive correlation on self-esteem, and has a significantly negative correlation on weight-loss intention, and self-esteem has a significantly positive correlation on weight-loss intention; meanwhile, self-efficacy and self-esteem can have an intermediary effect on body image and weight-loss intention, and “self-efficacy + self-esteem” also have a chained intermediary effect on body image and weight-loss intention.

### Limitation

There are still some limitations in this study. Firstly, this study is cross-sectional, meaning it cannot determine causal relationships between variables or establish temporal order. Compared to longitudinal data, this approach might lead to different estimates and conclusions. Therefore, future research could use longitudinal tracking studies to explore clearer causal relationships between variables and address these issues, aiding in further testing of the chain mediation model proposed in this study. Secondly, this study did not specifically screen college students for obesity (whether they are seeking treatment), depressive symptoms, or eating disorders, but it is hoped that future research can aim to address these limitations to provide a more comprehensive understanding. Finally, numerous factors influence undergraduates’ weight-loss intention. The effects of body image, self-efficacy and self-esteem on weight-loss intention are the only ones which are considered in this study. It is hoped that more external or psychological factors are supported to be used to explore the weight loss issue of undergraduates in the future, in purpose to help them develop healthy and confident lifestyles.

## Data Availability

The original contributions presented in the study are included in the article/supplementary material, further inquiries can be directed to the corresponding author.
